# A novel semiautomatic Chinese keywords instrument screening delirium based on electronic medical records

**DOI:** 10.1186/s12877-022-03474-w

**Published:** 2022-10-04

**Authors:** Ling Chen, Nan Li, Yuxia Zheng, Langli Gao, Ning Ge, Dongmei Xie, Jirong Yue

**Affiliations:** 1 Department of Geriatrics and National Clinical Research Center for Geriatrics, West China Hospital, Sichuan University, Chengdu, Sichuan Province China; 2 Department of Geriatrics, The Sixth People’s Hospital of Chengdu, Chengdu, Sichuan 610051 People’s Republic of China; 3 West China School of Nursing, Sichuan University, Chengdu, China

**Keywords:** Delirium, Keywords scale, Diagnosis, Screen, EMR, Reliability, Validity

## Abstract

**Background:**

Delirium is frequently unrecognized due to the absence of regular screening. In addition to validated bedside tools, the computer-assisted instrument based on clinical notes from electronic medical records may be useful.

**Aims:**

To assess the psychometric properties of a Chinese-chart-based keyword instrument for semiautomatically screening delirium using Natural language processing (NLP) based on clinical notes from electronic medical records.

**Methods:**

The patients were admitted to West China Hospital from January 2015 to December 2017. Grouping patients based on the medical notes, those with accessible physician documents but no nurse documents were classified as the physician & no-nurse (PNN) group, while those with accessible physician and nurse documents were classified as the physician & nurse (PN) group. The psychometric properties, test–retest reliability, internal consistency reliability (Cronbach's α), and criterion validity were calculated. Using receiver operating characteristic (ROC) analysis, the criterion validity of delirium was evaluated in comparison to the results of the Diagnostic and Statistical Manual of Mental Disorders, Fifth Edition.

**Results:**

A total of 779 patients were enrolled in the study. Their ages ranged from 65 to 103 years (82.5 ± 6.5), with men accounting for 71.9% of the total. A total of 312 patients had access to only physician documents in the physician & no-nurse (PNN) group, whereas 467 patients had access to both physician and nurse documents in the physician & nurse (PN) group. All 779 patients had a Cronbach's alpha of 0.728 in terms of reliability, with 100% test–retest reliability. The area under the ROC curve (AUC) values of the delirium screening instrument for criterion validity were 0.76 (all patients, *n* = 779), 0.72 (PNN, *n* = 312), and 0.79 (PN, *n* = 467), respectively.

**Conclusion:**

A delirium screening instrument composed of Chinese keywords that can be easily and quickly obtained from electronic medical records was developed, which improved delirium detection in older people.

**Trial registration:**

Not applicable.

**Supplementary Information:**

The online version contains supplementary material available at 10.1186/s12877-022-03474-w.

## Background

Delirium is an acute disturbance of attention, awareness, cognition, the sleep–wake cycle, and thought processes whose manifestation, severity, and duration tend to fluctuate. It is prevalent among older hospitalized patients, with incidence rates ranging from 14 to 56% [1], and is associated with longer hospital stays, higher healthcare costs, institutionalization, functional decline, cognitive impairment, and mortality [2]. Numerous bedside instruments have been developed and validated to screen delirium in elderly patients with a high degree of specificity and sensitivity [3], and they are widely utilized in clinical settings. However, delirium is frequently misdiagnosed in the clinic, particularly in the hypoactive subtype, where 76% of cases went unrecognized [4]. Based on the characteristics of acute onset and fluctuating course of delirium symptoms, daily screening is essential to detect and manage delirium to reduce in-hospital mortality [5].

Electronic medical records (EMRs), also referred to as electronic health records (EHRs), are widely used throughout the world for routine clinical investigation and management. EMRs store a variety of information, including patient demographics, medical and surgical histories, clinical notes, and more. Studies have been conducted to evaluate the accuracy of delirium symptoms documented in medical charts and manually generalized delirium characteristic keywords to be used as trigger words to detect delirium [6, 7]. Kuhn et al. [7] the data concerning delirium symptoms appeared more frequently in narrative notes, and there was a high degree of concordance between the physician and nursing narrative documentation [8]. Keywords include disorientation, agitation, altered level of consciousness, mental status, and a variety of other symptoms. These keyword methods are fragmented and require manually reviewing the entire chart and extracting keywords, with sensitivity ranging from 1.8% to 74% and specificity ranging from 65.1% to 100% [6, 7, 9, 10].

Natural language processing (NLP) is a computer-based approach that enables computers to comprehend what humans write and say. It has been widely utilized in the medical field to convert clinical narrative text into structured data [11, 12]. Wang at al. [12] that has demonstrated significant performance to aid clinicians in identifying clinically significant geriatric syndromes from clinical notes in electronic health records [13].

Therefore, a Chinese-chart-based keyword scale was developed to semiautomatically screen for delirium using NLP on clinical notes from electronic medical records. The purpose of this study was to assess the psychometric properties (reliability and validity) of this new instrument for detecting delirium.

## Methods

### Development of the keyword scale

A study team that met regularly for three months was established to develop this scale to identify delirium in elderly hospitalized patients. NG, a geriatrician, monitored the framework of the instrument and ensured the quality of the research; DMX, an advanced practice nurse and clinical research center controller, monitored the quality of the Delphi method. MZ, the primary study, reviewed the EMRs of 40 delirium patients and extracted the keywords. To determine the keywords, four research assistants, LC, XCP, TPL, and YLZ, reviewed twelve bedside scales and six chart methods for assessing delirium based on EMRs. Afterwards, data were collected, processed, and analysed. The initial keyword pool was derived from the following: twelve bedside scales involving delirium symptoms (CAM [14], 3D-CAM [15], CAM-CR [16], DSI [17], DOSS [18], DRS [19], DRS-R-98 [20], ICDSC [21], MDAS [22], MDS [23], NEECHAM [24], Nu-DESC [25]), six instruments assessing delirium based on EMRs, and 40 delirium patients' EMRs. In addition, the theoretical framework of the keyword scale was derived from the Diagnostic and Statistical Manual of Mental Disorders, Fifth Edition (DSM-V) [26] and the International Classification of Diseases, Tenth Revision (ICD-10) [27], which are currently accepted as the reference standard for delirium diagnosis [28]. Methods such as the Delphi method, the Analytic Hierarchy Process (AHP), and the item analyses of the classical test theory (CTT) were utilized during the development of the scale. The details have been previously published [29].

The initial scale had 59 items with 172 keywords, synonyms, and related words, related to delirium divided into 11 categories. This scale included particular medication, consultation (e.g., psychiatrist, neurologist, etc.), risk factors, delirium diagnosis, emotional lability, sleep–wake cycle disturbance, psychomotor disorder, inattention, altered level of consciousness, and other cognitive impairments including perceptual disturbances, disorientation, memory disorder, etc. The initial scale was then modified using the aforementioned methods and discussion among the study team. The category of risk factors, which had 32 items and 50 keywords, was removed for screening purposes rather than prediction. The formal keyword scale was then formed by 27 items with 122 keywords, with each item's score determined by Delphi weighting value assignment and ranging from 1.93 to 6.95, as shown in Additional file 1. Each keyword was rated as "no" and "yes" entries, with the keywords marked as "yes" scored as equal to the weight value. The total score ranged from 0 to 100 by adding up each item's score. A high score implies a high probability of delirium.

### Evaluation of the keywords by computer

A database was developed that contains the free text sections of clinical notes, such as daily nursing notes and daily physician progress notes, which are formed by the chief complaint, assessment, and physician's comments. Each data point corresponds to the hospitalization of a participant. The keyword frequency results were obtained by two data operators (NL, YFG) separately processing the database in R software and Microsoft Excel using NLP (word segmentation and word frequency statistics) embedded within an additional medical dictionary. The participant's score on the keyword scale was then determined. During the process, the sensitive information of the participants, such as their names, ID numbers, phone numbers, addresses, and other details, were concealed.

### Study population

The patients were admitted to West China Hospital, an academic medical center in southwest China, from January 2015 to December 2017. The inclusion criteria were as follows: (1) age ≥ 65 years, (2) available consent or surrogate consent, and (3) availability of relevant medical record information. Patients discharged within 48 h after admission were excluded. Demographical and clinical data were collected. To evaluate the psychometric properties with adequate statistical power, the sample size was set at 5–10 times the scale items, resulting in a sample of at least 160–320 patients. This study was approved by the Ethics Committee on Biomedical Research at the West China Hospital of Sichuan University.

### The psychometric properties of the instrument

#### Reliability: test–retest and internal consistency

Test–retest reliability and internal consistency reliability were both examined as reliability indicators. One computer engineer used R software to process the Chinese text database, and another computer engineer used Microsoft Excel to perform an independent analysis of the database. The two computer engineers were unaware of the delirium diagnosis. In addition, the internal consistency reliability of the scale was described using Cronbach's alpha coefficient [30].

#### Validity criterion

##### Reference standard diagnose for delirium

The geriatrician (JRY) independently determined that the patient had delirium based on DSM-V criteria and comprehensive face-to-face interviews conducted once daily while the patient was hospitalized. The assessment included a patient interview (standard psychiatric interview and mental status examination), family/caregiver interview, and medical staff interview. An expert panel including a geriatrician (JRY), a neurologist (STZ), a psychiatrist (LJJ), an anesthesiologist (JY), and a senior geriatric nurse (LLG)) adjudicated any doubted conclusions from the geriatrician (JRY). To avoid missing out on delirium, patients were monitored three times a day by trained nurses after admission. Furthermore, once a patient experienced an acute change in consciousness (dysphoria or drowsiness), a geriatrician evaluated him/her within 12 h. The expert panel was blinded to the results of the keyword scale, and the results of the DSM-V were managed by LLG.

##### Subgroup analysis

Due to limitations in the electronic medical records (EMRs) system, nursing records for a subset of patients were not accessible. Therefore, patients were divided into the physician & nurse (PN) and physician & no-nurse (PNN) groups based on whether nurse medical documents were involved, and the subgroup analysis evaluated the criterion validity separately for each group. Dementia and depression, two of the well-known differential diagnoses of delirium, may affect the accuracy of the tool; therefore, the criterion validity of dementia or depression was evaluated separately by subgroup analysis.

### The feasibility

Evaluating the applicability and acceptability of the new instrument in a clinical setting was planned. Here, the average time between the beginning of data collection and the completion of the final score, as well as the proportion of successful assessments, were used to evaluate feasibility.

### Statistical analysis

The patient characteristics were described using the mean (SD) for continuous variables and percentages for dichotomous and categorical variables for all participants, delirium patients, and patients without delirium. The test–retest reliability was determined by comparing keyword frequencies between two computer engineers. The internal consistency reliability was calculated by Cronbach's alpha coefficient. The performance of the new algorithm instrument was evaluated for criterion validity by calculating the area under the receiver operating characteristic curve (ROC, AUC). Following this, three thresholds were determined: 90% sensitivity, 90% specificity, and the maximum Youden Index. The sensitivity, specificity, positive likelihood ratio, and negative likelihood ratio were subsequently computed. All statistical data were analysed with SPSS version 23, and two-sided *p* values < 0.05 were considered statistically significant.

## Results

### Participant characteristics

**Fig. 1:**
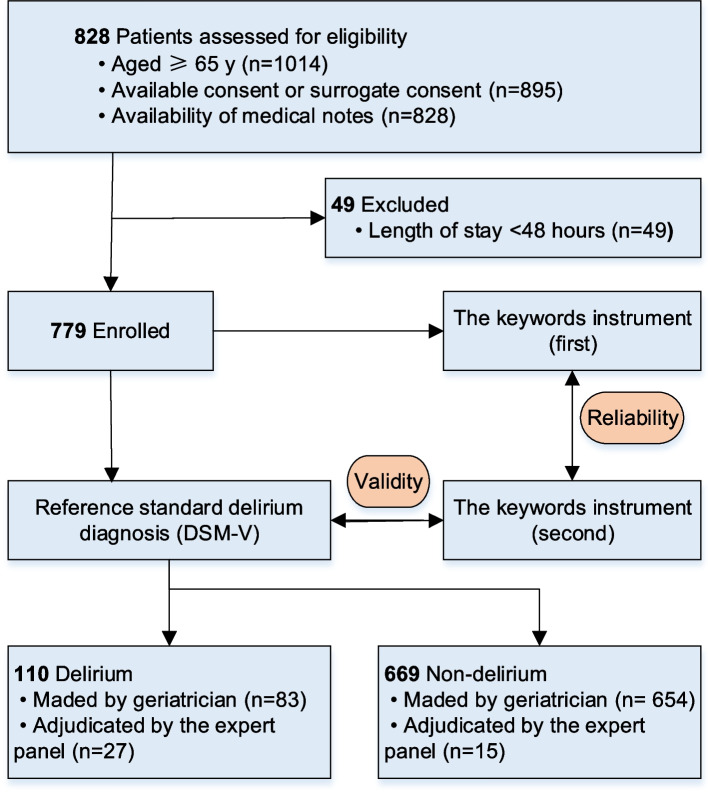
Flow diagram for the patients included in the study

As the flow diagram (Fig. 1), we involved 779 eligible patients and 779 physician medical notes, of which 467 had nurse medical documents concurrently (a group of nurse notes) and 312 did not have nurse medical documents (a group of nonnurse notes). The ages of the participants ranged from 65 to 103 years (mean = 82.5 years, S.D. 6.5 y), with a predominance of men (71.9%). As shown in Table 1, 6.2% (48/779) of the sample had a recognized or chart diagnosis of dementia, 4.1% (48/779) had depression, and 14.1% (110/779) had delirium that developed during hospitalization as diagnosed by DSM-V.

**Table 1:** Patient Characteristics

Characteristic	Patients (*N* = 779)	Delirium (*n* = 110)	Non-Delirium (*n* = 669)
Age, year, mean (SD)	82.8 (6.5)	82.4 (6.6)	85.2 (5.8)
Male Sex, n (%)	560 (71.9)	75 (68.2)	485 (72.5)
Ethnic, n (%)			
the Han nationality	767 (98.5)	110 (100)	657 (98.2)
minority	12 (1.5)	0	12 (1.8)
Length of stay, day, n (%)			
< 15	300 (38.5)	32 (29.1)	268 (29.1)
15–30	395 (50.7)	65 (59.1)	330 (49.3)
> 30	84 (10.8)	13 (11.8)	71 (10.6)
Dementia, n (%)	48 (6.2)	27 (24.5)	21 (3.1)
Depression, n (%)	32 (4.1)	6 (5.5)	26 (3.9)
Group by the medical chart, n (%)			
PN	312 (40.0)	70 (63.6)	242 (36.2)
PNN	467 (60.0)	40 (36.4)	427 (63.8)

*Abbreviation*: *SD* Standard deviation, *PN* Physician, and nurse, *PNN* Physician but no nurse

### The frequency of keywords

The top ten frequency keywords among 122 keywords on the scale for all patients are as follows: (1) poor spirit, 6422 times, (2) acceptable spirit, 2199 times, (3) bad sleep, 503 times, (4) poor sleep, 494 times, (5) nervous, 468 times, (6) fidgety, 355 times, (7) a little weak spirit, 353 times, (8) spirit not very well, 299 times, (9) drowsiness, 266 times, (10) nocturnal intermittent sleep, 204 times.

### The psychometric properties of the instrument

#### Reliability: test–retest and internal consistency

The instrument had a 100% test–retest reliability agreement. Data were obtained separately at different times using different technologies on different computers by blinded computer engineers in the 779 patients whose keywords frequency was 100% concordant. Then, it was determined that Cronbach's alpha for the entire scale was 0.728, which is an acceptable level for internal consistency reliability.

#### Validity criterion

**Fig. 2:**
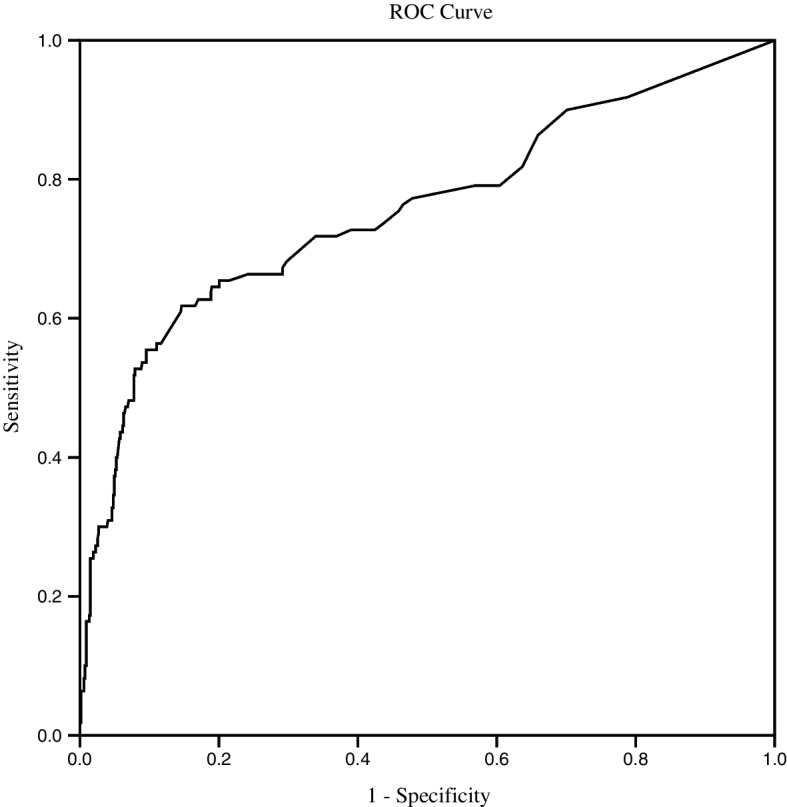
ROC curve of the instrument versus DSM-V for 779 patients

Analysis of the ROC curve revealed that the AUC value of the instrument identifying delirium relative to the DSM-V for 779 patients was 0.76 (95% CI: 0.69 to 0.81) (*P* < 0.001) (Fig. 2). The instrument score for 779 patients ranged from 0 to 55.86, with a mean score of 7.29. When the Youden Index was at its maximum, the cutoff value was 11.14 (Table 2). It identified delirium with a sensitivity of 61.8% and a specificity of 85.4%.

**Table 2:** The criterion validity of the instrument for identifying delirium at a different cutoff value

Cutoff	Youden Index	% (95% Confidence Interval)		Likelihood Ratio (95% Confidence Interval)	
		sensitivity	specificity	Positive	Negative
2.05	0.20	**90.0 (82.4—94.7)**	29.9 (26.5—33.5)	1.28 (1.19—1.39)	0.33 (0.19—0.59)
11.14	**0.47(max)**	61.8 (52.0—70.8)	85.4 (82.4—87.9)	4.22 (3.34—5.34)	0.45 (0.35—0.57)
12.27	0.45	55.5 (45.7—64.8)	**90.0 (87.4—92.1)**	5.54 (4.18—7.34)	0.50 (0.40—0.61)

Bold: Three thresholds determined methods (sensitivity 90%, specificity 90%, and maximum Youden Index)

##### Subgroup analysis

**Fig. 3:**
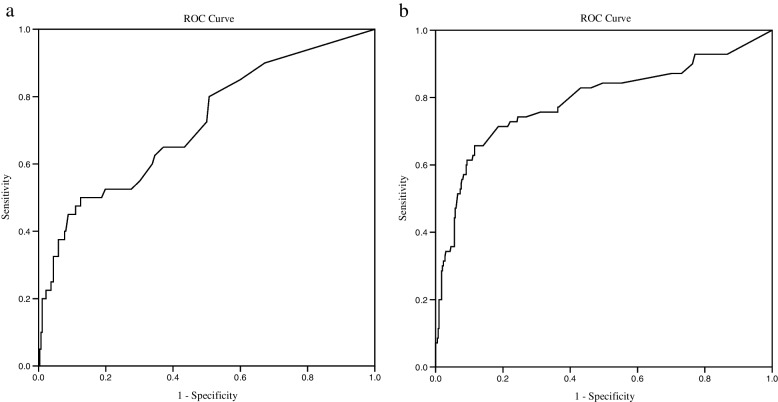
ROC curve for the PN (*n* = 312) **a** and PNN (*n* = 467) **b** groups

The AUC for the PN group (*n* = 312) was 0.72 (95% CI, 0.63 to 0.81) (*P* < 0.001), as illustrated in Fig. 3 (a). When the instrument's sensitivity was 90%, the threshold was 0.97, and its specificity was 32.7%, the positive likelihood ratio and the negative likelihood ratio were 1.04 and 0.22, respectively. Table 3 contains information regarding the validity of this and the other two thresholds.

**Table 3:** Subgroup analysis for the criterion validity of the instrument

Group	Cutoff	Youden Index	% (95% Confidence Interval)		Likelihood Ratio	
					**(95% Confidence Interval)**	
			**sensitivity**	**specificity**	**Positive**	**Negative**
**PN** (*n* = 312)	0.97	0.23	90.0 (75.4—96.7)	32.7 (27.2—38.7)	1.34 (1.17—1.53)	0.31 (0.12—0.79)
	9.03	0.38 (max)	50.0 (34.1—65.9)	87.5 (82.8—91.1)	4.00 (2.57—6.22)	0.57 (0.42—0.78)
	10.26	0.35	45.0 (29.6—61.3)	90.4 (86.2—93.5)	4.71 (2.85—7.77)	0.60 (0.46—0.81)
**PNN** (*n* = 467)	2.27	0.14	90.0 (79.9—95.5)	23.7 (19.6—28.2)	1.18 (1.07—1.30)	0.42 (0.21— 0.87)
	12.37	0.54 (max)	65.7 (53.3—76.4)	88.4 (84.8—91.3)	5.67 (4.12—7.81)	0.39 (0.28—0.54)
	13.76	0.52	61.4 (49.0—72.5)	90.7 (87.3—93.3)	6.60 (4.60—9.43)	0.43 (0.32—0.57)
**Dementia** (*n* = 47)	4.20	0.04	88.9 (69.7—97.1)	15.0 (4.0—38.9)	1.05 (0.83—1.53)	0.74 (0.15—3.75)
	22.00	0.38	48.1 (29.1—67.6)	90.0 (66.9—98.2)	4.81(1.22—18.98)	0.58 (0.40—0.84)
	25.00	0.4 (max)	44.4 (26.0—64.4)	95.0 (73.1—99.7)	8.88 (1.26—62.87)	0.58 (0.42—0.82)
**Non-dementia** (*n* = 732)	2.05	0.2	89.2 (79.9—94.6)	30.7 (27.2—34.4)	1.29 (1.17—1.40)	0.35 (0.19— 0.66)
	11.14	0.42 (max)	55.4 (44.1—66.2)	86.9 (84.0—89.4)	4.23 (3.21—5.58)	0.51 (0.40—0.65)
	11.82	0.38	48.2 (37.2—59.4)	90.1 (87.5—92.2)	4.89 (3.54—6.75)	0.57 (0.47—0.83)
**Depression** (*n* = 32)	9.27	0.01	66.7 (24.1—94.0)	34.6 (17.9—55.6)	1.02 (0.54—1.92)	0.96 (0.27—3.44)
	23.13	0.35 (max)	50.0 (13.9—86.1)	84.6 (64.3—95.0)	3.25 (0.97—10.85)	0.59 (0.26—1.33)
	27.99	0.01	16.7 (00.8—63.5)	84.6 (64.2—95.0)	1.08 (0.15—8.03)	0.98 (0.68—1.43)
**Non-depression** (*n* = 747)	2.05	0.22	91.3 (83.8—95.7)	30.9 (27.4—34.7)	1.32 (1.22—1.43)	0.28 (0.15—0.53)
	11.14	0.5 (max)	62.5 (52.4—71.6)	87.1 (84.2—89.5)	4.84 (3.77—6.22)	0.43 (0.34—0.55)
	11.82	0.47	56.7 (46.7—66.3)	90.0 (87.4—92.2)	5.70 (4.28—7.59)	0.98 (0.68—1.43)

*Abbreviation*: *PN* Physician, and nurse, *PNN* Physician but no-nurse

The AUC for the PNN group (*n* = 467) was 0.79 (95% CI, 0.72 to 0.86) (*P* < 0.001), as shown in Fig. 3 (b). The cutoff value for detecting the presence of delirium was 12.37 as the maximum Youden Index, with sensitivity (65.7%) and specificity (88.4%). The highest sensitivities for this tool were 88.9% and 66.7% in the dementia and depression groups, respectively. The other details are shown in Table 3.

### The feasibility

A computer completed the assessments in one minute, and 100% of the participants successfully assessed the instrument. It has excellent applicability and acceptability in the clinical setting for the assessment approach based on computer algorithms depending on the patients' EMRs.

## Discussion

### Comparison with other chart-based instruments

A one-minute instrument based on the keyword scale was developed to detect delirium by computer. This instrument demonstrated high test–retest reliability, acceptable internal consistency reliability, adequate criterion validity, and excellent applicability and acceptability during initial exploration. The score is a continuous variable, with higher scores indicating an increased probability of being diagnosed with delirium. Although the cutoff points that maximize sensitivity or specificity were excellent for these indicators, their opposites (specificity and sensitivity) were notably poor. The balanced cut-off point, on the other hand, had a low sensitivity (61.6%) for a screening test. As a result, three cut-off values were provided for the user to choose from. The accuracy can be improved significantly by incorporating the documents of the nurse. In comparison to DSM-V, its AUC was 0.75, and a significantly higher AUC was 0.79 in the charts with physicians' and nurses' notes. When the cutoff value was 12.37, the tool had relatively high sensitivity (65.7%) and specificity (88.4%) for identifying the presence of delirium. As a screening tool, it does not perform as well as the bedside scales. Yet, this new tool has excellent feasibility and applicability, which promises to achieve automated screening for promoting accurate and standardized management of delirium in hospitals.

With the development of EMRs, many researchers have attempted to identify delirium using EMRs and have achieved remarkable results. Professor Inouye developed the chart-based method (CHART-DEL) a few years ago [9]. The overall validity agreement between CHART-DEL and CAM was 82%, with a false positive rate of 26%. The research teams of Karla D adapted the CHART-DEL to CHART-DEL-ICU [9] for application in the intensive care unit (ICU). It took the independent raters approximately 28 min to divide the medical chart into five categories: no evidence, uncertain, possible, probable, and definite delirium. The AUC was 0.74 when the cut point of CHART-DEL-ICU was uncertain/possible/probable/definite. The AUC was reduced to 0.67 when delirium was probable/definite. Our instrument of validity property is generally consistent with the best performance of CHART-DEL-ICU and better in the group of physician and nurse notes (AUC = 0.79). The test–retest reliability (100%) was greater than that of the CHART-DEL-ICU, suggesting greater stability. In comparison to CHART-DEL-ICU, the current study instrument yields results quickly in just one minute, saving time and effort as part of the reform of the delirium assessment method.

Researchers recently developed a chart-based method for automated identification of the onset of delirium. The system identifies potential delirium episodes automatically based on the number of delirium prediction keywords recorded in the retrieval electronic rehabilitation database using a chart-based method with low to moderate accuracy [31]. Furthermore, its incident delirium criterion was classified by experts reviewing an electronic clinical database, with only 73.1% agreement between experts. A reference method is provided to develop the automated tool in the future.

### Clinical implications

Delirium evaluation is complicated for several reasons. First, delirium is a syndrome that manifests and fluctuates over a short period and is more commonly nocturnal. Second, it must be evaluated regularly. Third, older patients who were predisposed to delirium were assigned to each department. Fourth, delirium screening and diagnosis are based on symptoms rather than objective examination. The current delirium status in hospitalized patients is not ideological. Only 30% of delirium patients were identified as rarely performing daily delirium screening [4]. With a longer duration of delirium and a worse clinical prognosis, hypoactive delirium is more likely to be ignored. Recent studies have shown a significant increase in the documentation of delirium in discharge summaries, with 80.9% of patients having a delirium diagnosis. The symptom documentation in medical records for delirium is presumed to improve in the future, which will make our instruments more effective for screening delirium.

### The strengths and limitations of the instrument

The development process takes into consideration the goal of optimizing delirium detection at every stage, adopting the Delphi method and AHP scientifically. In addition, the likelihood ratios have demonstrated the multidimensional and scientific performance of the instrument. This instrument is a cost- and time-efficient, a semi-automatic screening tool for delirium patients that has excellent feasibility, filling a gap in designing EMRs-based semiautomatic delirium assessment system screening. The real-time computer assessment approach in EMRs for detecting delirium based on this tool is a promising development in further, updating the prevalence of delirium and providing researchers with to expand study cohorts.

The instrument has several limitations. First, the instrument was greatly influenced by the quality of medical notes. However, the psychometric properties of the instrument achieve desirable sensitivity and specificity, and we anticipated that it would improve in the future with more precise algorithms and higher-quality medical documents. Second, we conducted our entire investigation in Chinese. This instrument is only useful in general internal medicine wards in Chinese-speaking countries. In other words, it is applied to all Chinese medical record systems. Third, the acute onset and fluctuating characteristics of delirium were not included with many keywords, which is an area that needs more work in the future. However, due to the uniqueness of the Chinese language, identifying the keywords of acute onset or symptom fluctuation in Chinese is difficult. Fourth, there may be differences in how this instrument performs for various delirium types, which calls for more research into the topic. Finally, one of the limitations could be the gender imbalance, with 71.9% of the sample being male.

## Conclusions

We developed a novel instrument for computationally detecting delirium based on the keywords recorded in the physician & nurse' medical notes with an AUC of 0.79 (95% CI, 0.72 to 0.86). Provided a cost- and time-efficient tool for semi-automatic patient screening for delirium. The incidence and expression of delirium may be different in the population, contexts, and language, more prospective validation research is required.

## Supplementary Information

**Additional file 1.**

## Data Availability

The datasets generated and/or analysed during the current study are not publicly available due to the agreement of confidentiality but are available from the corresponding author on reasonable request.

## Abbreviations

3D-CAM: The 3-Minute Diagnostic Assessment for Delirium using the CAM algorithm
AHP: Analytic Hierarchy Process
AUC: The area under the ROC curve
CAM: The Confusion Assessment Method
CAM-CR: Confusion Assessment Method, Chinese reversion
CHART-DEL: A chart-based delirium detection tool
CHART-DEL-ICU: A chart-based delirium detection tool used in critically ill adults
CI: Confidence Interval
CTT: The Classical Test Theory
DOSS: Delirium Observation Screening Scale
DRS: Delirium Rating Scale
DRS-R-98: Delirium Rating Scale-revised-98
DSI: Delirium symptom interview
DSM-V: The fifth edition of the Diagnostic and Statistical Manual of Mental Disorders
EMRs: Electronic Medical Records
EHRs: Electronic Health Records
ICD-10: The International Classification of Diseases, Tenth Revision
ICDSC: Intensive Care Delirium Screening Checklist
ICU: Intensive Care Unit
MDAS: Memorial Delirium Assessment Scale
MDS: The Minimum Data Set
NEECHAM: The NEECHAM Confusion Scale
NLP: Natural language processing
Nu-DESC: The Nursing Delirium Screening Scale
PN: A Physician & Nurse group
PNN: A Physician & No-Nurse group
ROC: Receiver Operating Characteristic
SD: Standard Deviation
