# Supplementary material for: A novel semiautomatic Chinese keywords instrument screening delirium based on electronic medical records
Source: BMC Geriatr. 2022 Oct 4;22:779. doi: 10.1186/s12877-022-03474-w (PMC9531378; doi:10.1186/s12877-022-03474-w)
Supplement: Supplementary file 1 — Additional file 1. [file 12877_2022_3474_MOESM1_ESM.docx]

**A novel semiautomatic Chinese Keywords Instrument Screening Delirium based on Electronic Medical Records**

Ling Chen ^1, 2^, Nan Li ^1^, Yuxia Zheng ^1 3^, Langli Gao ^1 3^, Ning Ge ^1^, Dongmei Xie ^1 3^, Jirong Yue ^1^.

Dongmei Xie chenlinguse@163.com

English language version of the formal keyword scale

| **Category** | **Item** | **Keyword** | **Score** |
| --- | --- | --- | --- |
| Drugs | A1 | Haloperidol | 2.78 |
|  |  | Haldol |  |
|  | A2 | Olanzapine | 2.52 |
|  |  | Zyprexa |  |
| Consultation | B1 | Psychiatric consultation | 3.65 |
|  |  | Mental health center consultation |  |
|  |  | Psychological consultation |  |
|  |  | Consultation-liaison Psychiatry |  |
|  | B2 | Neurological consultation | 3.57 |
| Delirium | C1 | Delirium | 6.52 |
|  |  | Delirium state |  |
|  |  | Deliration |  |
|  | C2 | Encephalopathy | 4.04 |
|  |  | Disorder and organic psychosis |  |
|  |  | Psychological and behavioral abnormalities |  |
| Emotional disorder | D1 | Mistrust | 4.62 |
|  |  | Fidgety |  |
|  |  | Obvious Fidgety |  |
|  |  | Dysphoria |  |
|  |  | Irascible |  |
|  |  | Impatient |  |
|  |  | Mania |  |
|  |  | Vexed |  |
|  |  | Irritable |  |
|  |  | Irritability |  |
|  |  | Vigilance |  |
|  |  | Anxious |  |
|  |  | Anxious expression |  |
|  |  | Nervous |  |
|  |  | Intranquil |  |
|  |  | Aroused |  |
|  |  | Excited |  |
|  |  | Dynamic |  |
|  |  | Agitation |  |
|  |  | Agitated |  |
|  | D2 | Low spirits | 2.53 |
|  |  | Wretched |  |
|  | D3 | Mood swings | 3.24 |
|  |  | Emotionally unstable |  |
| Spirit state | E1 | Listlessness | 2.37 |
|  |  | Lethargy |  |
|  |  | Weakness |  |
|  | E2 | Poor spirit | 1.93 |
|  |  | Poor spirit status |  |
|  |  | Slightly poor spirit |  |
|  |  | Slightly worse mental condition |  |
|  |  | Spirit acceptable |  |
|  |  | Poor mental |  |
|  |  | Poor mental and appetite |  |
| Sleep | F1 | Sleep reversal | 3.59 |
|  | F2 | Nocturnal sleep disorder | 2.46 |
|  |  | Insomnia |  |
|  |  | Poor sleep |  |
|  |  | Poor sleep at night |  |
|  |  | Poor sleep quality |  |
|  |  | Bad sleep |  |
|  |  | Bad sleep at night |  |
|  |  | Intermittent sleep |  |
|  |  | Sleep intermittently at night |  |
|  | F3 | Slightly poor sleep | 2.17 |
|  |  | Slightly poor rest |  |
|  |  | Slightly worse sleep |  |
|  |  | Slightly poor night’s sleep |  |
|  |  | Slightly worse night’s sleep |  |
|  |  | Slightly poor night’s rest |  |
|  |  | Difficulty falling asleep |  |
|  |  | Difficulty falling asleep at night |  |
|  |  | Easy to wake |  |
|  |  | Easy to wake at night |  |
|  |  | Early awakening |  |
|  |  | Dreaminess |  |
|  |  | Dreaminess at night |  |
| Behavior and language disorder | G1 | Odd behavior | 3.99 |
|  |  | Groping action |  |
|  |  | Remove by self |  |
|  |  | Unpin by self |  |
|  |  | Aggressive behavior |  |
|  |  | Slapping |  |
|  |  | Swear |  |
|  | G2 | Uncooperative | 3.18 |
|  |  | Resistance |  |
|  |  | Poor adherence |  |
|  |  | Unable to cooperate |  |
|  |  | Refuse injection |  |
|  | G3 | Slowness of movements | 3.21 |
|  |  | Motion Slowly |  |
|  |  | Motionless |  |
|  |  | Stare |  |
|  | G4 | Speech abnormality | 4.43 |
|  |  | Nonsense |  |
|  |  | Babbled |  |
|  | G5 | Soliloquize | 3.99 |
|  |  | Bursts soliloquize |  |
|  |  | Murmur |  |
|  |  | Bursts moans |  |
|  |  | Sob |  |
|  |  | Less speech |  |
|  |  | Silence |  |
| Concentration impaired | H1 | Response obtusely | 6.95 |
| Consciousness alteration | I1 | Drowsiness | 5.77 |
|  |  | Lethargic sleep |  |
|  |  | Obnubilation |  |
|  |  | Unconsciousness |  |
|  |  | Adiaphoria |  |
|  |  | Consciousness change |  |
|  |  | Answer beyond the question |  |
|  |  | Irrelevant answer |  |
|  |  | Unable to answer |  |
|  |  | Unable to reply |  |
|  | I2 | Lack of consciousness | 5 |
|  |  | Haziness consciousness |  |
|  |  | Haziness Expression |  |
| Other cognitive disorder | J1 | Hallucination | 3.67 |
|  |  | Visual hallucination |  |
|  |  | Auditory hallucination |  |
|  | J2 | Incoherence of thinking | 3.58 |
|  | J3 | Delusion | 2.95 |
|  |  | Delusion of persecution |  |
|  | J4 | Recent memory impairment | 2.98 |
|  |  | Numeracy decline |  |
|  |  | Numeracy decreased |  |
|  | J5 | Disorientation | 4.32 |

*Note*: The English version of this instrument needs further studies to authenticate its applicability in English-speaking countries. The corresponding should be contacted for the verified Chinese version of the formal keyword scale.
